# Multidimensional deprivation and depressive symptom trajectories among single-person households in South Korea: an age-stratified longitudinal study

**DOI:** 10.1186/s12889-026-28102-9

**Published:** 2026-06-11

**Authors:** Taeyeon Kwon

**Affiliations:** Department of Social Welfare, Sun Moon University, Asan, Republic of Korea

**Keywords:** Single-person households, Depressive symptoms, Multidimensional deprivation, Social relationships, Latent growth modeling, Life-course perspective

## Abstract

**Background:**

The rapid growth of single-person households represents a major demographic shift with significant implications for public mental health. Adults living alone may be particularly vulnerable to depression due to reduced social support and heightened exposure to various forms of disadvantage. While the link between socioeconomic status and mental health is well-documented, there is a need for longitudinal evidence that accounts for the multidimensional nature of deprivation across different life stages. This study examined the age-stratified associations between multidimensional deprivation and depressive symptom trajectories among adults living alone.

**Methods:**

Data were drawn from six waves (2018–2023) of the Korea Welfare Panel Study (KOWEPS), including 2,094 adults (aged ≥ 20) in single-person households. Depressive symptoms were assessed repeatedly using the CESD-11. Multidimensional deprivation was operationalized across six domains: basic living, housing, social security, employment/economic status, social relationships, and healthcare. Latent growth modeling (LGM) was employed to estimate initial levels and rates of change in depressive symptoms. To identify age-specific patterns, analyses were conducted separately for young, middle-aged, and older adults.

**Results:**

Depressive symptoms among single-person households showed a significant upward trajectory over time. In age-stratified analyses, the associations between multidimensional deprivation domains and depressive symptoms varied across life stages. Basic living deprivation was more strongly associated with depressive symptoms among young adults, whereas economic and healthcare deprivation were more strongly associated among middle-aged adults. For older adults, housing and healthcare deprivation showed stronger associations with depressive symptom levels. Notably, social relationship deprivation was consistently associated with both baseline levels and trajectories of depressive symptoms across all age groups.

**Conclusions:**

The longitudinal progression of depressive symptoms in single-person households is associated with multidimensional deprivation, with varying impacts across the life course. These findings suggest that public health interventions for those living alone should move beyond a one-size-fits-all approach and instead implement age-specific, multidimensional strategies. Particular focus should be placed on addressing social connectivity and healthcare access, which remain important factors in mental health across all stages of adulthood.

## Introduction

The rapid increase in single-person households represents a major demographic transformation with important implications for public mental health. Individuals living alone are more likely to experience reduced social support, economic vulnerability, and limited access to resources, all of which have been linked to elevated risks of depressive symptoms [1–4].

In South Korea, this shift is particularly pronounced, with single-person households now accounting for over 34% of all households and representing one of the fastest-growing household types among OECD countries [5–7]. This demographic trend reflects broader societal changes, including population aging, delayed marriage, and shifts in family structures [8, 9]. As this population continues to grow, understanding the multidimensional determinants of their mental health has become an important public health priority.

A substantial body of research has demonstrated that deprivation is a key determinant of mental health outcomes [10–14]. Rather than being limited to income-based or unidimensional measures, deprivation is increasingly conceptualized as a multidimensional construct encompassing various domains of disadvantage, including material conditions, access to services, and social relationships [15–18].

In this study, deprivation is conceptualized as a multidimensional construct that extends beyond traditional socioeconomic status (SES) frameworks. Consistent with the Alkire–Foster approach, this framework allows for the inclusion of non-economic domains—such as social relationship deprivation—without compromising conceptual coherence [16, 17]. By explicitly distinguishing between material conditions (e.g., housing, employment, and social security) and relational or psychosocial resources, this approach avoids conflating material deprivation with social isolation, a distinction that is particularly important for individuals living alone, whose vulnerabilities often span multiple domains [3, 19].

This multidimensional perspective is especially relevant in contemporary societies, where social and relational deficits may independently contribute to mental health risks even in the absence of severe material deprivation [2, 4].

Previous research has examined the relationship between disadvantage and depressive symptoms using a range of methodological approaches, including longitudinal designs [10–12]. These studies consistently highlight that socioeconomic disadvantage is associated with poorer mental health outcomes; however, they often rely on aggregated or unidimensional indicators, which may obscure the distinct contributions of different deprivation domains [13, 14].

In particular, relatively less attention has been paid to how specific domains of multidimensional deprivation shape trajectories of depressive symptoms within the context of single-person households [20, 21]. Moreover, the meaning and impact of deprivation are likely to vary across the life course, as age-related social roles and circumstances influence how individuals experience and respond to disadvantage [17, 22].

For example, deprivation in early adulthood may be closely linked to labor market instability and challenges in social integration, whereas in later life it may reflect accumulated disadvantage, social isolation, and increasing healthcare needs [8, 23]. These differences suggest that the pathways linking deprivation to mental health across the life course are not uniform but instead vary depending on life-stage–specific contexts.

Despite the growing body of longitudinal research, relatively limited attention has been given to age-specific patterns of these associations [12, 20]. This gap is particularly important for individuals living alone, as their exposure to different forms of deprivation may vary substantially across the life course.

Against this background, the present study examines how multidimensional deprivation is associated with trajectories of depressive symptoms among individuals living alone, using longitudinal data from the Korea Welfare Panel Study (2018–2023). By focusing on single-person households, employing a multidimensional operationalization of deprivation, and examining age-specific patterns across the life course, this study aims to provide a more nuanced understanding of age-specific mental health inequalities in this rapidly growing population.

## Methods

### Participants

**Fig. 1:**
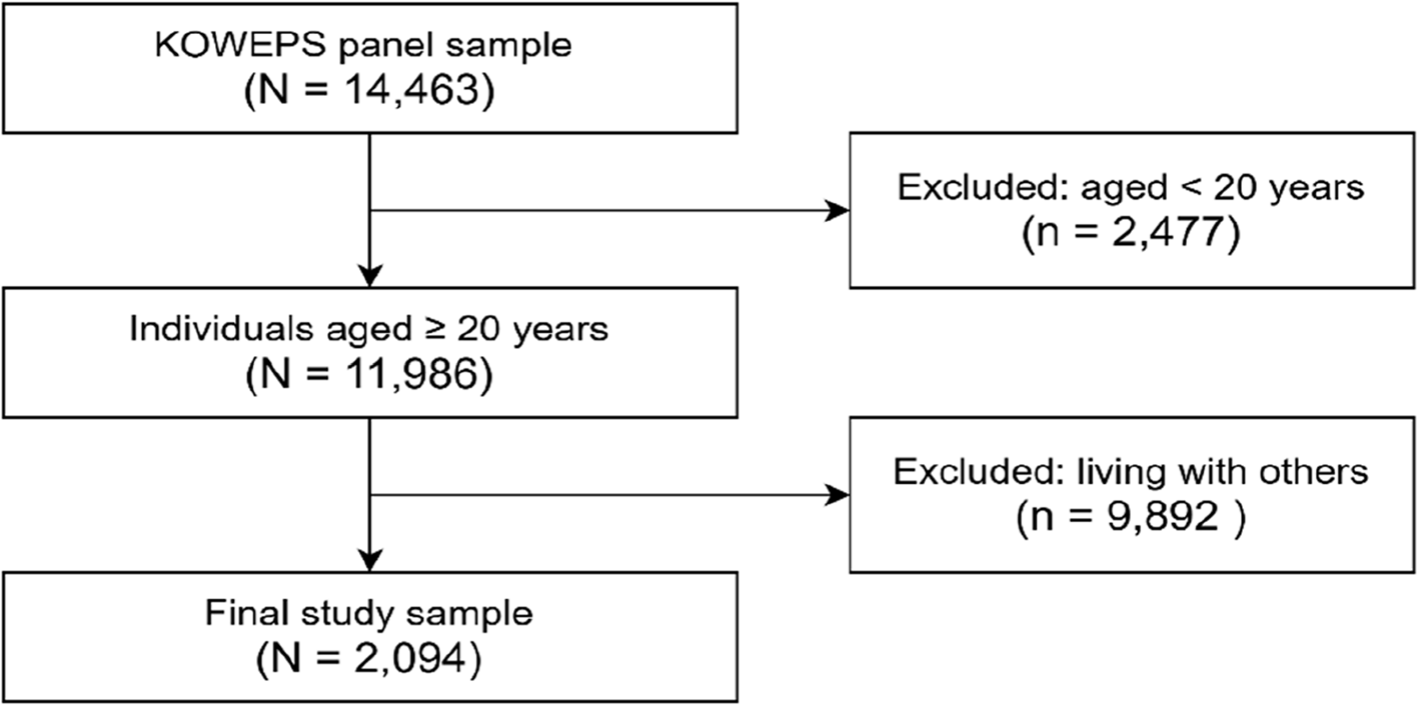
Flow diagram of sample selection from the KOWEPS

This study used data from the nationally representative Korea Welfare Panel Study (KOWEPS), conducted annually since 2006 by the Korea Institute for Health and Social Affairs and the Social Welfare Research Institute of Seoul National University. KOWEPS provides comprehensive information on households and individuals, including social service needs, healthcare utilization, economic and demographic characteristics, income, and mental health. The initial sampling employed a probability-based selection of 7,072 households from a sampling frame of 30,000 households, yielding responses from 14,463 individuals. For the present study, six waves of data (2018–2023) were used, focusing on variables that were consistently measured across survey waves. The analytic sample was restricted to respondents aged 20 years or older who lived in single-person households, resulting in a final sample of 2,094 individuals. The sample selection process is illustrated in Fig. 1.

This study used secondary data from the KOWEPS, a publicly available dataset. The data are fully anonymized, and no identifiable personal information is accessible. Therefore, this study did not require institutional review board approval under national ethical guidelines. Informed consent was obtained from all participants by the original survey agency at the time of data collection.

### Measures

This secondary analysis utilized validated Korean versions of instruments employed in prior studies.

#### Depressive symptoms

Depressive symptoms were assessed using the 11-item Center for Epidemiologic Studies Depression Scale (CESD-11), which has been validated for use in the Korean general population [24]. Each item was rated on a four-point Likert scale ranging from 0 (rarely or none of the time) to 3 (most or all of the time). Item scores were summed to create a total CESD-11 score at each wave, with higher scores indicating greater severity of depressive symptoms. Possible total scores ranged from 0 to 33. Internal consistency was high across the six waves, with Cronbach’s α values ranging from 0.900 to 0.916.

#### Multidimensional deprivation

Table 1 summarizes the items classified within each domain of multidimensional deprivation. The multidimensional deprivation framework used in this study was conceptually informed by prior theoretical and empirical research on deprivation and mental health. Classical approaches, particularly Townsend’s framework of relative deprivation, emphasize that disadvantage arises not only from income poverty but also from limited access to material resources, adequate living conditions, and opportunities for social participation [15]. Building on this perspective, subsequent research has conceptualized deprivation as a multidimensional construct encompassing several domains relevant to health outcomes [13, 14]. In the present study, multidimensional deprivation was conceptualized as encompassing both material conditions (e.g., housing, employment, and social security) and relational or psychosocial resources (e.g., social relationships). This approach extends beyond traditional socioeconomic status (SES) frameworks by explicitly incorporating relational dimensions that are particularly salient for individuals living alone. The multidimensional deprivation framework was conceptually developed by the authors and operationalized using indicators available in the KOWEPS dataset. Accordingly, deprivation was categorized into six domains: (1) basic living conditions, (2) housing, (3) social security, (4) employment and economic conditions, (5) social relationships, and (6) health and medical access. These domains were selected to capture key conditions associated with mental health among individuals living alone. Most deprivation indicators were derived directly from the KOWEPS dataset and coded as binary variables indicating whether the respondent experienced a specific form of deprivation (1 = experienced deprivation, 0 = not experienced). For relational deprivation, satisfaction with family relationships and social relationships was measured using a five-point Likert scale (1 = very dissatisfied to 5 = very satisfied). These variables were recoded into dichotomous indicators, with dissatisfaction (1–2) coded as 1 and all other responses (3–5) coded as 0. For each domain, item scores were summed to construct domain-specific deprivation scores, with higher scores indicating greater levels of deprivation. Detailed descriptions of domains and items are presented in Table 1. Importantly, in South Korea, participation in public pension and health insurance systems is mandatory. Therefore, non-enrollment may reflect socioeconomic vulnerability, such as unstable employment or exclusion from formal labor markets, rather than voluntary choice. This contextual interpretation is important for understanding the meaning of social security deprivation in this study.

**Table 1:** Multidimensional deprivation domains and items

Domain	Question
Basic living domain	Experience of running out of food due to financial difficulties but not having money to buy more; inability to afford a balanced diet due to financial hardship; failure to pay utility bills on time; disconnection of electricity, phone, or water services due to unpaid bills.
Housing domain	Experience of moving due to unpaid rent or overdue rent for more than two months; inability to afford heating during winter; whether the dwelling is above ground level; whether it is a durable permanent structure; adequacy of soundproofing, ventilation, lighting, and heating facilities; exposure to environmental hazards (noise, vibration, odor, pollution); safety from natural disasters; exclusive use of kitchen and bathroom facilities.
Social security domain	Enrollment in public pension plan; enrollment in health insurance.
Employment/Economic domain	Employment status; ability to cover minimum living costs; experience of working in a hazardous environment.
Social domain	Satisfaction with family relationships; satisfaction with social relationships.
Health/Medical domain	Experience of being unable to visit a hospital due to lack of money; subjective health status.

All items were coded or reverse-coded so that higher values consistently indicate greater multidimensional deprivation (0 = no deprivation, 1 = deprivation)

####  Control variables

Following previous research on socioeconomic determinants of depression [10], age, gender (0 = female, 1 = male), education (0 = middle school or lower, 1 = high school or higher), and average monthly income were included as control variables. These variables were included to adjust for potential confounding influences but are not central to the study’s primary research questions.

### Data analysis

Descriptive statistics, including means, standard deviations, skewness, and kurtosis, were calculated using SPSS version 23. Latent growth modeling (LGM) was conducted using full-information maximum likelihood (FIML) estimation in AMOS version 23. Under the FIML framework, all available data were used, and individuals with at least one valid observation were included in the analysis.

The analysis proceeded in two stages. First, an unconditional growth model was estimated to examine overall trajectories of depressive symptoms over time among individuals living in single-person households. Second, baseline multidimensional deprivation domains measured in 2018 were entered as time-invariant predictors of both the intercept and slope factors of depressive symptoms.

Model fit was evaluated using multiple indices, including the chi-square statistic (χ²), Tucker–Lewis Index (TLI), Comparative Fit Index (CFI), and Root Mean Square Error of Approximation (RMSEA). Following commonly accepted criteria, CFI values of 0.90 or higher and RMSEA values of 0.08 or lower were considered indicative of acceptable model fit [21–23]. Although a formal post-hoc power analysis was not conducted, the sample size was considered sufficient based on prior latent growth modeling studies, which have suggested that sample sizes in the range of 200 or more are generally adequate for estimating growth parameters and modeling longitudinal trajectories in multi-wave data [25, 26]. Given the complexity of latent growth modeling and the use of six repeated waves, the present sample size was considered adequate for stable parameter estimation.

## Results

### Descriptive statistics

Table 2 presents the distribution of respondents across the six survey waves by age group. The final analytic sample included 2,094 individuals living in single-person households, consisting of 234 young adults, 321 middle-aged adults, and 1,539 older adults at baseline. Across the six waves (2018–2023), the overall panel retention rate remained above 74%, indicating relatively stable participation over time.

**Table 2:** Distribution of the analytic sample across survey waves and age groups

Wave	Young adults	Middle-aged adults	Older adults	Total
2018	234	321	1539	2,094
2019	230	314	1453	1,997
2020	211	303	1366	1,880
2021	179	224	1347	1,750
2022	158	194	1287	1,639
2023	141	183	1230	1,554

Table 3 summarizes the sociodemographic characteristics and key variables of the analytic sample. Most individuals living in single-person households were aged 65 years or older, female, had a middle school education or lower, and reported relatively low levels of annual disposable income, with a large proportion earning less than 2 million Korean won per year.

**Table 3:** Descriptive statistics for sociodemographic and key variables *(N* = 2,094)

Variable		Frequency	Percentage (%)	
Age	Aged 20–44 years	234	11.2	
	Aged 45–64 years	321	15.3	
	Aged 65 years or older	1,539	73.5	
Education	middle school or lower	1,515	72.3	
	High school or higher	579	27.7	
Sex	Male	521	24.9	
	Female	1,573	75.1	
Income	< 1 million KRW	842	40.2	
	1–1.99 million KRW	754	36.0	
	2–2.99 million KRW	251	12.0	
	3–3.99 million KRW	128	6.1	
	≥ 4 million KRW	119	5.6	

**Variable**		**Mean(SD)**	**Skewness**	**Kurtosis**
Wave 1 depressive symptoms		5.81(5.51)	1.18	1.32
Wave 2 depressive symptoms		5.82(5.53)	1.15	1.01
Wave 3 depressive symptoms		5.91(5.73)	1.16	0.96
Wave 4 depressive symptoms		6.23(5.77)	1.15	1.23
Wave 5 depressive symptoms		6.51(5.75)	1.04	0.73
Wave 6 depressive symptoms		5.94(5.57)	1.20	1.15
Basic living deprivation		0.13(0.43)	3.65	4.35
Housing deprivation		1.58(1.18)	2.48	2.22
Social security deprivation		1.45(0.62)	-0.68	-0.51
Employment/Economic deprivation		0.67(0.49)	-0.49	-1.19
Social deprivation		0.14(0.39)	2.73	3.10
Health/Medical deprivation		0.38(0.49)	0.69	-1.11

### Unconditional model of depressive symptoms in single-person households

Before estimating the conditional growth models, an unconditional latent growth model was estimated to examine the trajectory of depressive symptoms over time and to determine the appropriate functional form of change. Model comparison indicated that the linear growth model fit the data significantly better than the no-growth model (Δχ² = 54.78, Δdf = 6, *p* < .001). Although the quadratic model produced a slightly lower chi-square value, the chi-square difference test indicated that the inclusion of a quadratic term did not significantly improve model fit (Δχ² = 2.39, Δdf = 3, *p* > .05). In addition, the quadratic model showed poorer fit indices (RMSEA = 0.150, TLI = 0.835, CFI = 0.835) compared with the linear model (see Table 4). Therefore, the linear specification was retained as the most parsimonious representation of change in depressive symptoms over time. Given the relatively small number of observation waves in the present study, the linear model also provides a stable and interpretable representation of change.

**Table 4:** Comparison of unconditional latent growth models

Model	χ^2^	df	RMSEA	TLI	CFI
No-growth	244.741	27	0.075	0.929	0.929
Linear	189.959	21	0.062	0.947	0.947
Quadratic	187.572	18	0.150	0.835	0.835

Model comparison was based on the chi-square difference test

The unconditional model indicated significant variability in both the intercept and slope of depressive symptoms. The estimated mean intercept was 5.934 (SE = 0.110), with a significant variance of 15.866 (SE = 0.801, *p* < .001), suggesting substantial individual differences in baseline levels of depressive symptoms. The mean linear slope was 0.130 (SE = 0.029), with a significant variance of 0.416 (SE = 0.052, *p* < .001), indicating that depressive symptoms increased over time on average and that individuals differed in their rates of change. These results indicate considerable heterogeneity in both the initial levels and trajectories of depressive symptoms among individuals living alone. In addition, the covariance between the intercept and slope was negative and statistically significant, suggesting that individuals with higher baseline levels of depressive symptoms tended to exhibit smaller increases in symptoms over time.

### Multidimensional deprivation and depressive symptom trajectories

The conditional latent growth model incorporating multidimensional deprivation as time-invariant covariates showed good overall model fit (χ² = 287.173, *p* < .001; TLI = 0.940; CFI = 0.973; RMSEA = 0.042). Standardized parameter estimates from the conditional latent growth model are presented in Table 5.

**Table 5:** Results of the conditional latent growth model for CESD

Deprivation		Outcome	B	S.E.	C.*R*.	β	*p*
Basic living deprivation	➔	CESD intercept	1.026	0.238	4.307	0.118	0.000
	➔	CESD slope	− 0.067	0.072	− 0.930	− 0.047	0.352
Housing deprivation	➔	CESD intercept	0.029	0.134	0.216	0.011	0.829
	➔	CESD slope	− 0.054	0.040	-1.350	− 0.161	0.177
Social security deprivation	➔	CESD intercept	0.623	0.209	2.975	0.102	0.003
	➔	CESD slope	− 0.105	0.056	1.853	− 0.100	0.064
Employment/Economic deprivation	➔	CESD intercept	0.843	0.371	2.269	0.097	0.023
	➔	CESD slope	− 0.037	0.067	− 0.544	− 0.038	0.586
Health/Medical deprivation	➔	CESD intercept	2.794	0.207	13.483	0.345	0.000
	➔	CESD slope	− 0.173	0.052	-3.326	− 0.136	0.000
Social deprivation	➔	CESD intercept	2.614	0.252	10.380	0.262	0.000
	➔	CESD slope	− 0.300	0.076	-3.960	− 0.174	0.000

Several multidimensional deprivation domains were associated with depressive symptom trajectories. Deprivation in the basic living domain (β = 0.118, *p* < .001), social security domain (β = 0.102, *p* < .01), and employment/economic domain (β = 0.097, *p* < .05) was associated with the intercept of depressive symptoms only. In contrast, housing deprivation was not associated with either the intercept or slope of depressive symptoms.

Social deprivation and health/medical deprivation were associated with both the intercept and slope of depressive symptoms. Social deprivation was positively associated with the intercept (β = 0.262, *p* < .001) and negatively associated with the slope (β = −0.174, *p* < .001). Similarly, health/medical deprivation was positively associated with the intercept (β = 0.345, *p* < .001) and negatively associated with the slope (β = −0.136, *p* < .001).

**Fig. 2:**
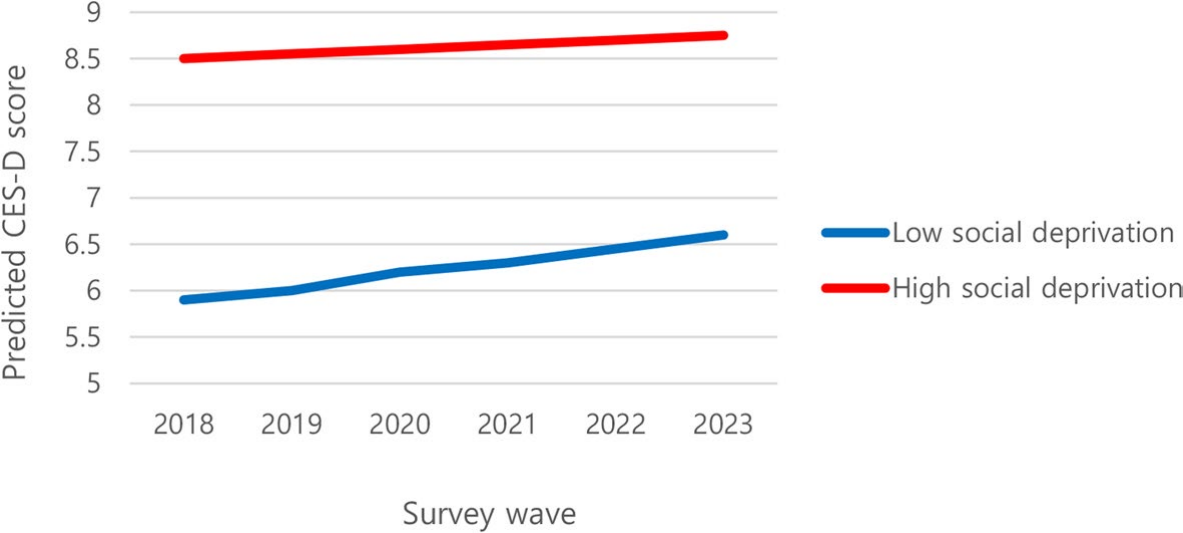
Predicted trajectories of depressive symptoms by social deprivation

To facilitate interpretation of these growth parameters, predicted trajectory plots were generated. Figure 2 presents the predicted trajectories of depressive symptoms by level of social deprivation.

## Age-stratified results

**Fig. 3:**
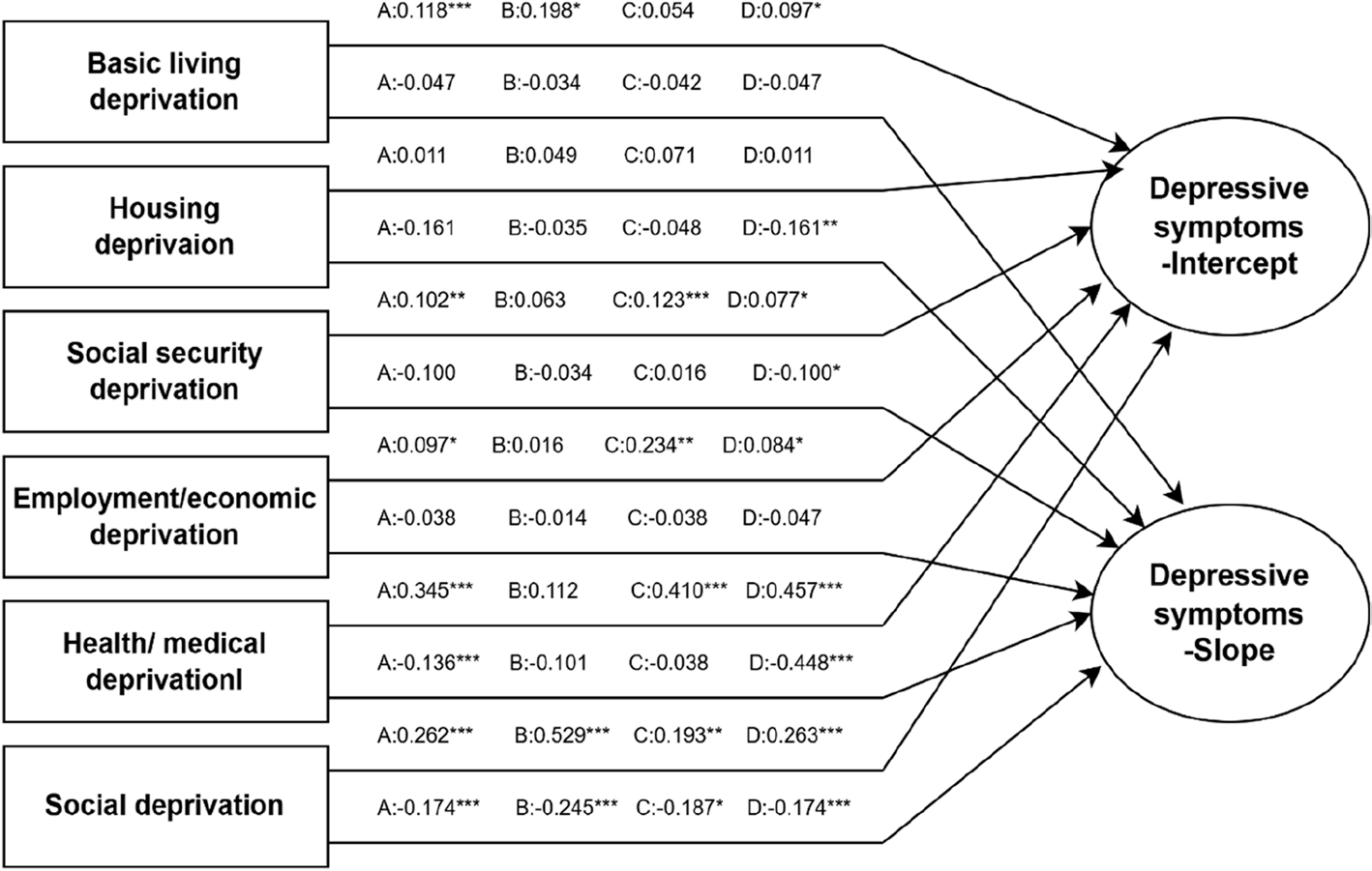
Results of the age-stratified models showing standardized path coefficients for associations between multidimensional deprivation and depressive symptom trajectories. Note. Standardized path coefficients are presented for the total sample and age-stratified groups. A = total adult sample; B = young adults; C = middle-aged adults; D = older adults. Control variables (age, gender, education, and income) were included in the model but are not shown for clarity

Age-stratified models were estimated to examine associations between multidimensional deprivation and depressive symptom trajectories within each age group (young adulthood, middle adulthood, and older adulthood). Figure 3 displays path coefficients for the total sample and for each age group. In the figure, A represents the total adult sample, B represents young adults, C represents middle-aged adults, and D represents older adults.

For young adults living alone, deprivation in the basic living domain was associated with the intercept of depressive symptoms (β = 0.198, *p* < .05). Social deprivation was associated with both the intercept (β = 0.529, *p* < .001) and slope (β = −0.245, *p* < .001) of depressive symptoms.

For middle-aged individuals living alone, health/medical (β = 0.410, *p* < .001), employment/economic (β = 0.234, *p* < .01), and social security deprivation (β = 0.123, *p* < .001) were associated with the intercept of depressive symptoms. Social deprivation was associated with both the intercept (β = 0.193, *p* < .01) and slope (β = −0.187, *p* < .05).

Among older adults living alone, health/medical (intercept: β = 0.457, *p* < .001; slope: β = −0.448, *p* < .001), social security (intercept: β = 0.077, *p* < .05; slope: β = −0.100, *p* < .05), and social deprivation (intercept: β = 0.263, *p* < .001; slope: β = −0.174, *p* < .001) were associated with depressive symptoms. Housing deprivation was associated with the slope (β = −0.161, *p* < .01). In contrast, employment/economic (β = 0.084, *p* < .05) and basic living deprivation (β = 0.097, *p* < .05) were associated with the intercept.

## Discussion

The present study examined longitudinal trajectories of depressive symptoms among adults living in single-person households and investigated how multidimensional deprivation is associated with both baseline levels and changes in depressive symptoms across adulthood. Using nationally representative longitudinal data, the findings indicate that depressive symptoms among individuals living alone tended to increase over time in the study sample. By integrating a multidimensional deprivation framework with a life-course perspective, this study provides new empirical evidence on how multidimensional deprivation shapes both the baseline level and trajectories of depressive symptoms among individuals living alone.

Importantly, different patterns of association were observed across different stages of adulthood, suggesting that individuals living alone should not be treated as a homogeneous population. Rather, trajectories of depressive symptoms within this group appear to be shaped by both life-course stage and broader social conditions.

The sample composition of this study reflects the demographic structure of single-person households in South Korea, where a large proportion of individuals living alone are older adults. National statistics indicate that population aging is a major driver of the rapid growth of single-person households in the country [5, 6]. In this context, the observed increase in depressive symptoms over time may partly reflect life-course processes associated with aging among individuals living alone, including age-related health decline, cumulative exposure to socioeconomic hardship, and changes in social roles [8, 21, 27].

A key finding of this study concerns the role of social deprivation. Social deprivation showed associations with both the baseline level and the trajectory of depressive symptoms across age groups. Notably, the positive association with the intercept combined with the negative association with the slope suggests that individuals experiencing higher levels of social deprivation begin with elevated depressive symptoms and that these disparities persist over time rather than narrowing. These findings suggest that limited social connectedness may function as a persistent vulnerability factor for individuals living alone, influencing not only the initial level of depressive symptoms but also how symptoms evolve over time. This interpretation is consistent with previous research indicating that social isolation and limited social networks are closely associated with depressive symptoms and psychological distress [3, 4, 19]. Because individuals living alone lack immediate sources of emotional and instrumental support within the household, reduced opportunities for social interaction may increase vulnerability to depression. In this context, social connectedness may serve as an important protective factor for mental health among individuals living alone [4, 28].

Other domains of multidimensional deprivation demonstrated distinct patterns within each stage of adulthood. Among young adults living alone, deprivation in the basic living domain was primarily associated with higher baseline depressive symptoms. Early adulthood is often characterized by unstable employment, financial precarity, and transitions in housing and family roles [29, 30]. Previous research has shown that financial hardship and socioeconomic disadvantage are associated with increased psychological distress across adulthood [11, 12]. When individuals in this stage live alone, these challenges may be experienced without the buffering effects of shared household resources.

Among middle-aged adults living alone, employment/economic, health/medical, and social security deprivation were associated primarily with higher baseline depressive symptoms. This pattern suggests that mental health in midlife may be particularly sensitive to the combined effects of socioeconomic and health-related vulnerability. Midlife is often characterized by increasing work-related pressures, financial responsibilities, and emerging health concerns [31, 32]. For individuals living alone, these challenges may be intensified by the absence of shared household resources. In the South Korean context, non-enrollment in mandatory public pension or health insurance systems is unlikely to reflect voluntary choice and may instead indicate unstable employment, exclusion from formal labor markets, or broader socioeconomic vulnerability. Such exclusion may thus compound the psychological burden of midlife. Among older adults, health/medical and social security deprivation were associated with both baseline levels and changes in depressive symptoms over time. This finding aligns with previous research suggesting that declining physical health and limited social resources are important determinants of mental health in later life [27, 28]. In addition, housing deprivation was uniquely associated with changes in depressive symptoms among older adults in this study. Housing conditions may become particularly relevant in later life because older adults spend more time in their residential environments and may have fewer opportunities to relocate or modify their housing situations. Previous research has also shown that inadequate housing environments are associated with psychological distress and poorer mental health outcomes [23].

These findings underscore that the meaning and impact of deprivation are context-dependent and vary across stages of the life course.

Overall, the findings contribute to the literature on socioeconomic inequalities and mental health in several ways. First, by examining depressive symptom trajectories using longitudinal data, this study moves beyond cross-sectional associations and highlights how socioeconomic conditions shape mental health patterns over time among individuals living alone. Second, the age-stratified analyses demonstrate that the relationship between multidimensional deprivation and depressive symptoms varies across different stages of adulthood, suggesting that individuals living alone should not be treated as a homogeneous group. Third, by adopting a multidimensional perspective on socioeconomic deprivation, this study shows that mental health among individuals living alone is influenced not only by economic conditions but also by broader structural and relational contexts. In particular, the consistent association between social deprivation and depressive symptom trajectories underscores the importance of social connectedness as a key determinant of mental health among people living alone [3, 4]. Relatively few longitudinal studies have simultaneously examined multidimensional deprivation and trajectories of depressive symptoms across different stages of adulthood among individuals living alone.

Although the present study focuses on South Korea, the findings may have broader relevance for societies experiencing rapid population aging and an increasing prevalence of single-person households. Cross-national comparative research would be valuable for examining whether similar patterns emerge under different welfare regimes and institutional contexts [3, 7]. As living alone becomes more common worldwide, understanding how socioeconomic conditions interact with life-course processes will be increasingly important for addressing emerging mental health inequalities.

Importantly, the findings should be interpreted as associative rather than causal relationships. Although longitudinal data were used to examine depressive symptom trajectories, multidimensional deprivation was measured at baseline, and reverse or bidirectional relationships between deprivation and depressive symptoms cannot be ruled out. Future research incorporating time-varying measures of socioeconomic conditions would provide a more comprehensive understanding of how changes in socioeconomic environments influence mental health trajectories among individuals living alone.

Taken together, these findings highlight the importance of adopting a life-course–oriented perspective when addressing mental health inequalities among individuals living in single-person households. Policies aimed at reducing mental health disparities should therefore consider not only individual risk factors but also broader socioeconomic conditions that shape vulnerability across different stages of adulthood.

### Policy and practice implications

The findings of this study have important implications for both clinical practice and community-based mental health services targeting adults living alone. At the population level, the results highlight the importance of integrating mental health screening and social risk assessment into existing public health and primary care systems serving individuals in single-person households [17, 33].

First, assessment and early identification strategies should consider life-course differences in sources of vulnerability. For young adults living alone, routine screening for depressive symptoms in primary care and community settings may help identify individuals experiencing economic precarity and unmet basic needs. For middle-aged adults, integrated assessment of mental health, employment-related stress, and health insurance status may be particularly relevant. For older adults, systematic depression screening combined with evaluations of housing conditions and medical needs may improve early detection of mental health risks [27, 28].

Second, interventions addressing social isolation should be incorporated into clinical and community mental health services. Strengthening referral pathways between outpatient mental health services and community-based support programs—such as peer support groups, social prescribing initiatives, and group-based activities—may help mitigate depressive symptoms among individuals living alone [34, 35].

Third, mental health social workers and community mental health professionals may play an important role in coordinating care across service sectors. Integrating psychosocial assessment with clinical treatment planning can facilitate continuity of care across medical, mental health, and social service systems [17].

Finally, low-intensity and stepped-care approaches may be appropriate for adults living alone who experience persistent social and health-related deprivation. This implication is supported by the finding that social deprivation was consistently associated with both baseline levels and trajectories of depressive symptoms across age groups, while health/medical deprivation showed particularly strong associations among middle-aged and older adults. These findings suggest that not all individuals living alone require the same level of intervention. A stepped-care model could begin with routine screening, psychoeducation, social prescribing, and community-based support, with referral to more intensive psychological or psychiatric care when depressive symptoms persist or worsen [36].

## Conclusion

As the prevalence of single-person households continues to increase globally, this study provides new evidence on how multidimensional deprivation is associated with depressive symptom trajectories among adults living alone. Using longitudinal data and an age-stratified analytic approach, the findings demonstrate that depressive symptoms are shaped by multiple domains of deprivation, with distinct patterns observed across different stages of adulthood.

The results highlight that depression among adults living alone cannot be understood solely as an individual or clinical condition but should be considered within a broader context of cumulative socioeconomic disadvantage. In particular, social deprivation emerged as a key factor associated with both baseline levels and trajectories of depressive symptoms, underscoring the importance of social connectedness for mental health. Other domains of multidimensional deprivation showed distinct patterns of association across stages of adulthood, indicating that the factors linked to depressive symptoms vary across the life course. Overall, these findings emphasize the importance of integrating mental health care with attention to broader socioeconomic conditions that shape vulnerability among individuals living alone. A life-course–oriented perspective may therefore provide a useful framework for addressing the growing mental health challenges faced by single-person households in aging societies.

## Data Availability

The original data presented in the study are openly available in https://www.koweps.re.kr and may be accessed upon reasonable request.

## Abbreviations

OECD: Organization for Economic Co-operation and Development
KOWEPS: Korea Welfare Panel Study
CESD: Center for Epidemiologic Studies Depression Scale
SES: Socioeconomic Status
LGM: Latent Growth Modeling
FIML: Full-Information Maximum Likelihood
CFI: Comparative Fit Index
TLI: Tucker–Lewis Index
RMSEA: Root Mean Square Error of Approximation
